# Prospecting for Energy-Rich Renewable Raw Materials: Sorghum Stem Case Study

**DOI:** 10.1371/journal.pone.0156638

**Published:** 2016-05-27

**Authors:** Caitlin S. Byrt, Natalie S. Betts, Hwei-Ting Tan, Wai Li Lim, Riksfardini A. Ermawar, Hai Yen Nguyen, Neil J. Shirley, Jelle Lahnstein, Kendall Corbin, Geoffrey B. Fincher, Vic Knauf, Rachel A. Burton

**Affiliations:** 1 Australian Research Council Centre of Excellence in Plant Cell Walls, School of Agriculture, Food and Wine, University of Adelaide, Urrbrae, South Australia, Australia; 2 Arcadia Biosciences, Davis, CA, United States of America; Iowa State University, UNITED STATES

## Abstract

Sorghum vegetative tissues are becoming increasingly important for biofuel production. The composition of sorghum stem tissues is influenced by genotype, environment and photoperiod sensitivity, and varies widely between varieties and also between different stem tissues (outer rind vs inner pith). Here, the amount of cellulose, (1,3;1,4)-β-glucan, arabinose and xylose in the stems of twelve diverse sorghum varieties, including four photoperiod-sensitive varieties, was measured. At maturity, most photoperiod-insensitive lines had 1% w/w (1,3;1,4)-β-glucan in stem pith tissue whilst photoperiod-sensitive varieties remained in a vegetative stage and accumulated up to 6% w/w (1,3;1,4)-β-glucan in the same tissue. Three sorghum lines were chosen for further study: a cultivated grain variety (*Sorghum bicolor* BTx623), a sweet variety (*S*. *bicolor* Rio) and a photoperiod-sensitive wild line (*S*. *bicolor* ssp. *verticilliflorum* Arun). The Arun line accumulated 5.5% w/w (1,3;1,4)-β-glucan and had higher *SbCslF6* and *SbCslH3* transcript levels in pith tissues than did photoperiod-insensitive varieties Rio and BTx623 (<1% w/w pith (1,3;1,4)-β-glucan). To assess the digestibility of the three varieties, stem tissue was treated with either hydrolytic enzymes or dilute acid and the release of fermentable glucose was determined. Despite having the highest lignin content, Arun yielded significantly more glucose than the other varieties, and theoretical calculation of ethanol yields was 10 344 L ha^-1^ from this sorghum stem tissue. These data indicate that sorghum stem (1,3;1,4)-β-glucan content may have a significant effect on digestibility and bioethanol yields. This information opens new avenues of research to generate sorghum lines optimised for biofuel production.

## Introduction

Plants in the *Sorghum* genus are an important source of chemical energy in the form of carbohydrates for animals, humans and biofuels [1, 2]. Cultivated sorghums all belong to *S*. *bicolor* subsp. *bicolor* and there are five races: bicolor, caudatum, durra, guinea and kafir [3–6]. Two subspecies within *S*. *bicolor* (*drummondii* and *verticilliflorum* (previously *arundinaceum*) are wild weedy relatives of cultivated sorghums [7, 8]. For example, *S*. *bicolor* subsp. *verticilliflorum* (Steud., De Wet ex Wiersema & J. Dahlb.; previously classified as *S*. *arundinaceum*) is a widely distributed common grass found in weedy patches along roadsides in northern Australia [9, 10]. Germplasm diversity marker analysis has revealed that *S*. *bicolor* subsp. *bicolor* is closely related to wild weedy sorghums and that genetic variation in this subspecies is high [11].

Cultivated varieties of sorghum are commonly grouped according to their end uses, for example, grain sorghum (food and feed), forage sorghum, sweet sorghum (for sugar production) and bioenergy sorghum [12, 13]. There are notable differences in the relative carbon partitioning and morphology between these groups: grain varieties produce large heads of grain rich in starch; sweet sorghums produce a tall, sugar-rich stem; and bioenergy and forage sorghums produce a large amount of vegetative biomass [14].

The composition of the cell wall in the stem tissue varies between genotypes [15, 16] and even within a single stem: the outer rind comprises different tissues than does the inner pith [17–19]. Understanding how sorghum stem cell wall composition affects biomass digestibility is important for improving forage quality and for developing high yielding bioenergy or biofuel crops [1, 20–23]. In general, the amount of sucrose, cellulose and non-cellulosic polysaccharides in mature sorghum biomass is affected by genotype, environmental conditions and photoperiod sensitivity [24, 25].

The most abundant cell wall component in sorghum vegetative tissues is usually cellulose, which is a polymer of (1,4)-β-linked glucosyl residues. Cellulose is synthesised at the plasma membrane by cellulose synthase A (CESA) proteins, which function as subunits of a rosette-shaped complex. Loss of function of CESA proteins tends to result in weak stems and irregular or thin cell walls [20]. Dicotyledonous plants have type I cell walls and the non-cellulosic polysaccharide constituents are pectins and xyloglucans whereas grasses such as sorghum have type II cell walls which contain heteroxylans (arabinoxylan and glucuronoarabinoxylan) and (1,3;1,4)-β-glucan and only a small amount of pectin [20]. In grass heteroxylans, the (1,4)-β-xylan chain is commonly substituted with α-arabinofuranosyl (Ara*f*) residues attached most commonly at the *O*-3 position of the xylosyl residues, but also at the *O*-2 position and occasionally at both *O*-2 and *O*-3 [26]. Ara*f* substituents can be esterified with hydroxycinnamic acids such as ferulic acid and *p*-coumaric acid [20]. (1,3;1,4)-β-Glucan consists of unsubstituted, unbranched chains of (1,3)- and (1,4)-β-glycosyl residues. The random but non-repeating (1,3)-linkages prevent molecules from aggregating, thereby precluding crystallisation and allowing for limited solubility [27]. Cellulose synthase-like genes, such as *CslF* and *CslH*, are involved in synthesising (1,3;1,4)-β-glucan [28, 29].

The amount and type of lignin (a complex polyphenolic cell wall polymer) influences biomass digestibility, and has been a long-standing barrier to efficient glucose release from woody plant materials [30]. Previous studies in sorghum have investigated reducing biomass recalcitrance by reducing the lignin content [31, 32]. Alternatively, altering the monolignol composition or the ferulate cross linking of lignin to arabinoxylan have been proposed as solutions [33, 34]. Examples also exist where a decrease in cellulose reduces biomass recalcitrance [35]. However, we are yet to understand the relative contribution to recalcitrance that can be attributed specifically to non-cellulosic polysaccharides such as arabinoxylan or (1,3;1,4)-β-glucan.

Genetic variation for cell wall composition in cultivated sorghum has been investigated [15, 17] but a large pool of untapped diversity exists in other species and subspecies [2]. Previously, a population derived from a high-biomass sweet sorghum ‘Rio’ and a grain sorghum ‘BTx623’ was used to investigate the genetic basis of leaf and stem biomass composition [15]. Quantitative trait loci (QTL) for structural and non-structural carbohydrate yields were identified that co-localised with QTL for height, flowering time and stand density-tillering. In sorghum, the composition of leaf and stem structural carbohydrates are under separate genetic control; sorghum stem composition is more heritable than leaf composition and contributes more to the total plant biomass than does leaf tissue [15, 36]. Theoretical data from this population predicted that biomass yield would have a greater effect on ethanol yield than biomass composition, but the authors suggest that future work should investigate greater genetic diversity for biomass traits [15].

The highest biomass yields reported for sorghum are from photoperiod-sensitive hybrids [12]. Many wild and forage sorghums are photoperiod-sensitive and remain in a prolonged vegetative state without flowering until the day length drops below 12h 20 min [37]. Breeding strategies have removed photoperiod sensitivity traits to allow sorghum to mature and produce grain in the longer day lengths of temperate areas [37]. In the context of biofuel production from vegetative biomass, the use of photoperiod-sensitive sorghum hybrids with long vegetative growth results in higher biomass yields, however there is limited information about the composition of biomass produced when photoperiod-sensitive lines remain in a vegetative state [12].

In the present study, cell wall composition in the stem of twelve diverse sorghum lines was investigated with a focus on BTx623, Rio, and a partially photoperiod-sensitive wild *S*. *verticilliflorum* line ‘Arun’, to explore how differences in amounts and distribution of cell wall components affect stem digestibility.

## Results

### Variation in Biomass Traits in Diverse *Sorghum* Genotypes

**Fig 1:**
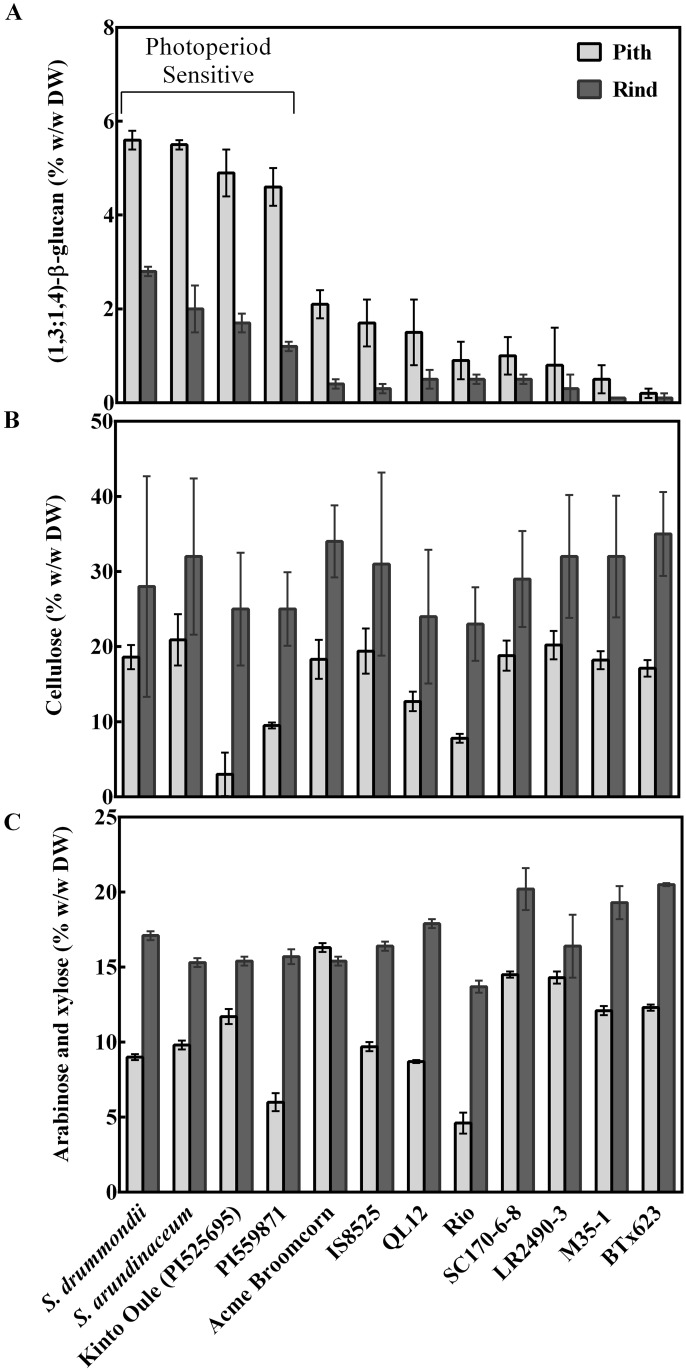
Stem cell wall composition for diverse *Sorghum* genotypes. The amount of (**a**) (1,3;1,4)-β-glucan, (**b**) cellulose and (**c**) arabinose plus xylose in % (w/w) in stem tissues of various genotypes. Mean and standard error for n = 3 biological replicates; three technical replicates per assay. The photoperiod-sensitive lines did not flower with the exception of ssp. *verticilliflorum*, which is a partially photoperiod-sensitive line. The remaining photoperiod-insensitive lines had flowered and reached maturity at harvest.

Twelve genetically diverse sorghum lines were grown in controlled greenhouse conditions. At maturity eight photoperiod-insensitive lines were harvested (129 d after planting). Maturity was delayed in photoperiod-sensitive lines. One partially photoperiod-sensitive line was harvested at maturity (159 d after planting), while the remaining photoperiod-sensitive lines remained in a vegetative state and were harvested 248 d after planting. Pith and rind tissue from the third internode above the base of each plant stem was harvested and the amounts of (1,3;1,4)-β-glucan, cellulose, arabinose and xylose were quantified (Fig 1, S1 Table). The amount of mannose, ribose, rhamnose, glucuronic acid and galacturonic acid released from acid-hydrolysed polysaccharides in the pith and rind samples were determined: mannose, ribose and rhamnose accounted for 0–0.1% w/w, glucuronic acid accounted for 0.2–0.5% w/w and galacturonic acid accounted for 0.1–0.9% w/w.

Large differences were observed between pith and rind composition. The (1,3;1,4)-β-glucan content in the pith was, on average, 2.4% w/w for the 12 genotypes studied compared with 0.9% w/w in the rind (Fig 1a). The rind contained more cellulose, with 29% w/w in rind vs 17% w/w in the pith (Fig 1b), and more arabinose and xylose moieties, with 17% w/w in rind vs 11% w/w, than the pith (Fig 1c). Notably, the stems of photoperiod-sensitive varieties, three of which did not flower, contained approximately five times more (1,3;1,4)-β-glucan at 5.2% w/w in the pith compared with 1.9% w/w in the rind than photoperiod-insensitive varieties (1.1% w/w in pith, 0.3% w/w in rind; Fig 1a).

### Carbon Partitioning in Sorghum Plants

**Fig 2:**
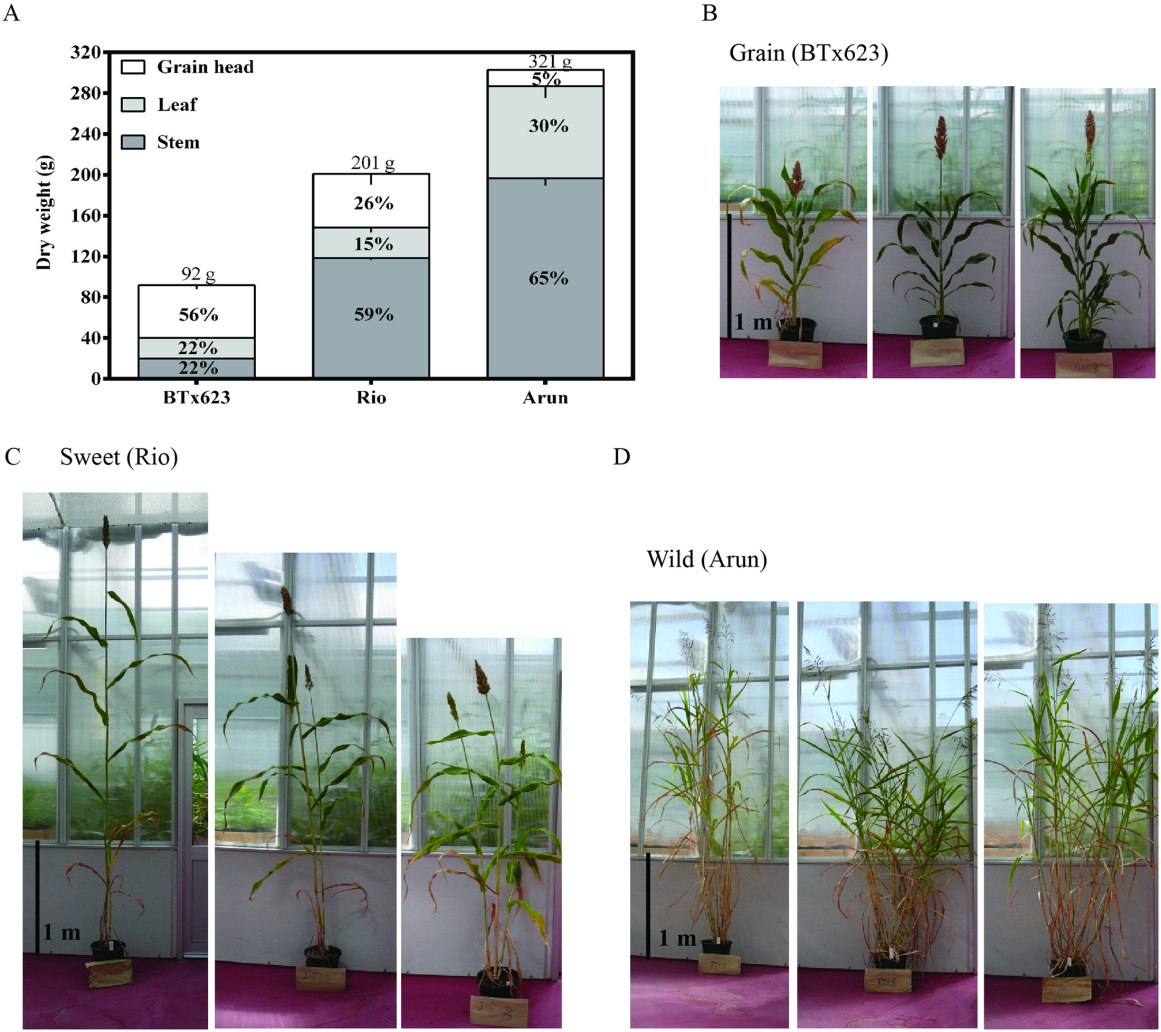
Diversity in morphological and physiological traits in wild, sweet and grain sorghum lines. Percentage contribution of grain head, leaf and stem to plant dry weight (mean; with SEM indicated by vertical line; n = 3 biological replicates) in mature (**b**) grain (*S*. *bicolor* ‘BTx623’), (**c**) sweet (*S*. *bicolor* ‘Rio’) and (**d**) wild (*S*. *bicolor* ssp. *verticilliflorum* ‘Arun’) sorghum lines. Rio and BTx623 plant were photographed 129 d after planting and Arun 159 d after planting.

To further assess variation in diverse sorghum lines, the morphology of three types of sorghum, a grain (*S*. *bicolor* BTx623), a sweet (*S*. *bicolor* Rio) and a wild (*S*. *bicolor* ssp. *verticilliflorum* Arun), was investigated. Plant height, tiller diameter and number, wet and dry biomass, water content of whole plants and harvest index for leaf, stem and grain head tissues was recorded at maturity (Fig 2, S2 Table). The three lines displayed many architectural and morphological differences and varied in their biomass production (Fig 2). The sweet and wild lines were similar in height (2.5 m), which was approximately double the height of the grain line. The wild line had thinner tillers but produced many more per plant (13) than the other two lines (1–2 tillers per plant). Water content was similar for all varieties, ranging from 63–68%.

Overall biomass production varied considerably. The average dry biomass yield of the wild line (321 g) was 1.6 times greater than the yield of the sweet line and three times greater than that of the grain line (S2 Table). The harvest index, which describes how plants allocate biomass to leaves, stems and grain head, also varied considerably. Stem tissue in the wild and sweet lines accounted for 59–65% of the biomass, whereas in the grain line, the grain head constituted 56% of the dry biomass (Fig 2a).

**Fig 3:**
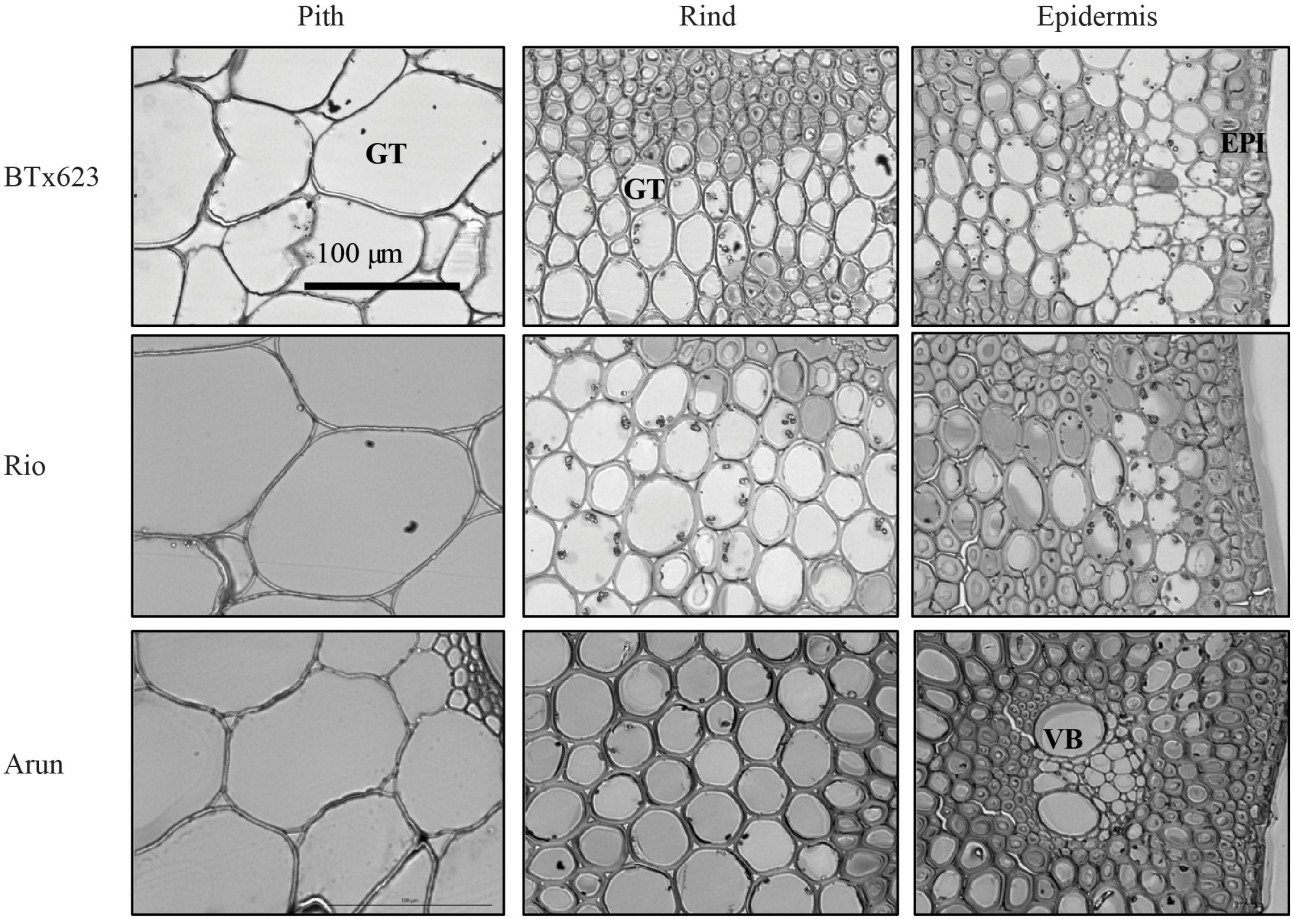
Cell wall characteristics in stems of grain, sweet and wild sorghum lines. Transmission electron micrographs of transverse sections of pith, rind and epidermal tissues for BTx623, Rio and Arun. Images are all of the same scale, bar indicates 100 μm. Ground tissue (GT), vascular bundle (VB), epidermis (EPI).

Images of stem sections that are representative of the pith, rind and epidermal tissues for each line were captured (Fig 3) but reveal no obvious differences in the size of the cells or thickness of the cell walls in the different genotypes. The cell wall composition of each line was therefore analysed to explore biochemical differences.

### Cell Wall Composition in Sorghum Stems

**Fig 4:**
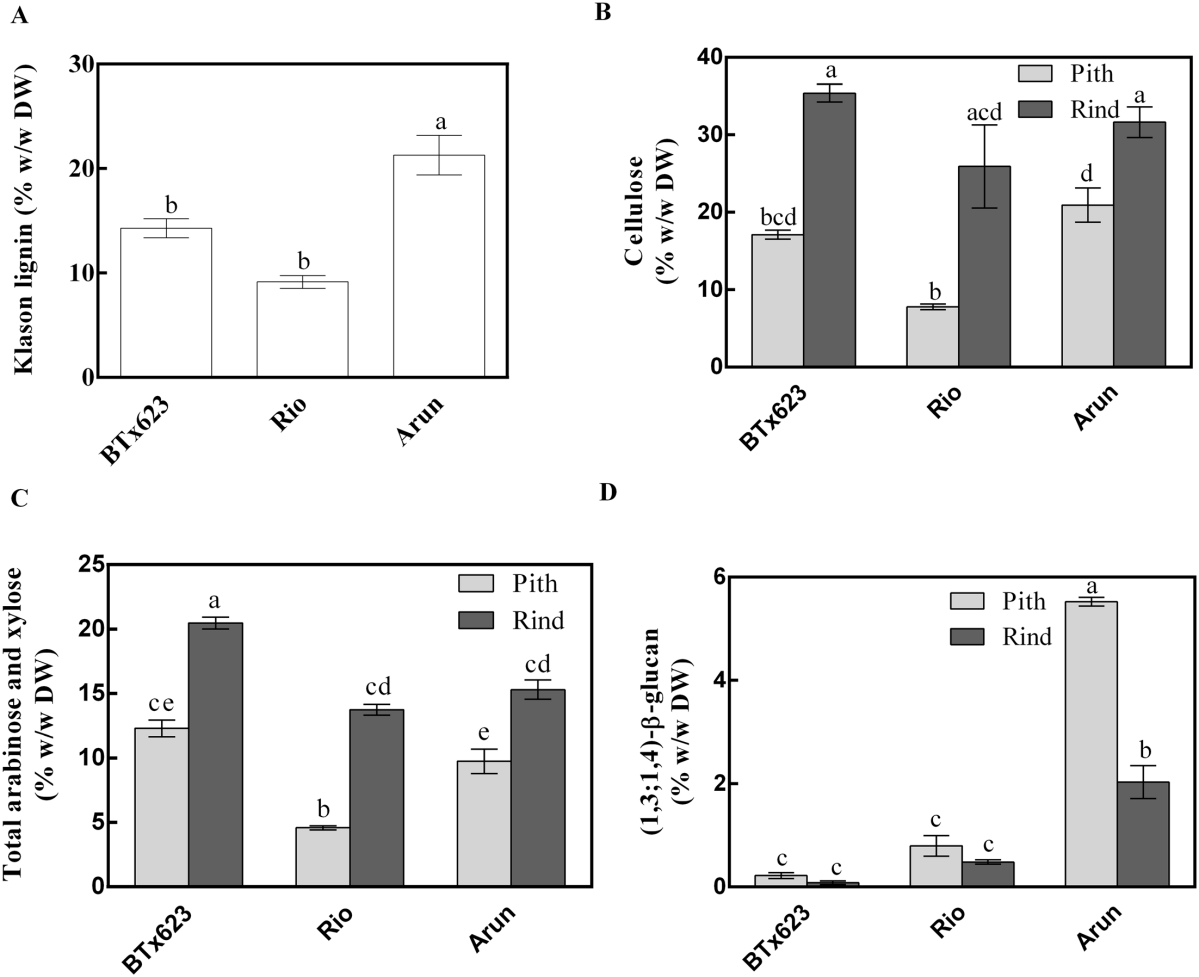
Cell wall components in grain, sweet and wild sorghum lines. (**a**) Klason lignin, (**b**) Updegraff cellulose, (**c**) total arabinose plus xylose, and (**d**) (1,3;1,4)-β-glucan in sorghum stem sections. Mean and standard error of n = 3 biological replicates, three technical replicates per assay. Different letters indicate statistical differences (ANOVA details in S3 Table).

**Fig 5:**
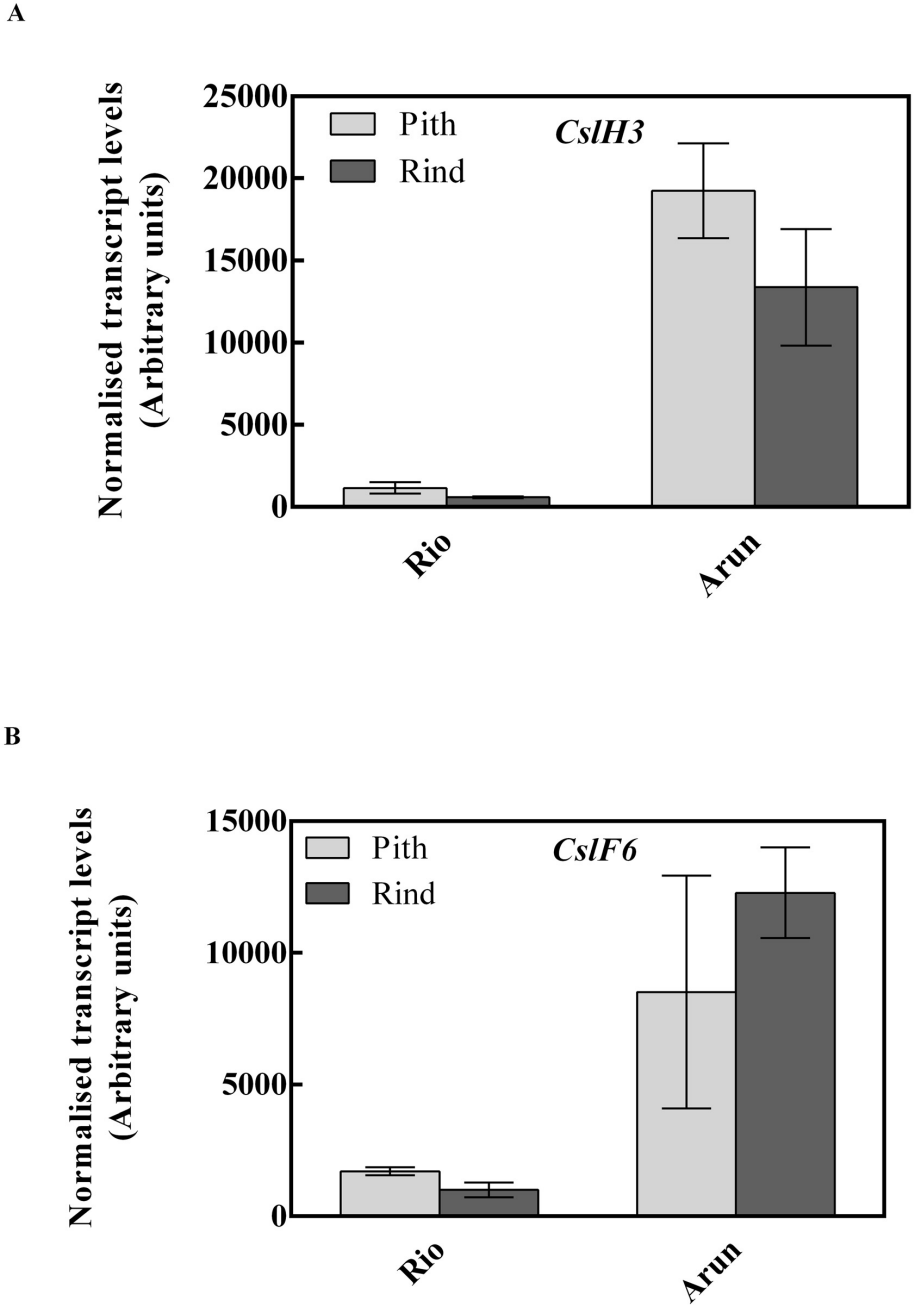
Transcript analysis in sorghum stem sections. Transcript profile of (a) *SbCslH3* and (**b**) *SbCslF6* in pith, rind and whole stem tissues of Rio and Arun. The transcript levels of these genes in the stem of mature BTx623 were reported previously [38]. Mean and standard errors n = 3 biological replicates. Data are normalised to control genes (S4 Table).

We compared the amount of lignin, acid-insoluble cellulose, arabinose plus xylose and (1,3;1,4)-β-glucan in whole stem or pith and rind tissues of BTx623, Rio and Arun (Fig 4). The amount of arabinose and xylose released from acid-hydrolysed polysaccharides was used as an estimation of the amount of arabinoxylan in the samples, although it is acknowledged that arabinose will also be a constituent of the relatively small amounts of type I and II arabinogalactans in the tissues. The Klason (acid-insoluble) lignin content of whole stem samples was highest in the wild line, with significantly lower amounts in the grain and sweet varieties (Fig 4a). As with the twelve diverse genotypes, pith contained less cellulose and arabinoxylan than rind tissue (Fig 4b–4d). The grain variety and wild line contained the most cellulose and lignin, while the sweet variety contained the least. Again, (1,3;1,4)-β-glucan was much higher in the wild, partially photoperiod-sensitive line (Arun) compared with the two photoperiod-insensitive lines (Fig 4d). Consistent with this finding, when transcript levels of two genes previously reported to be involved in (1,3;1,4)-β-glucan synthesis, namely *SbCslF6* and *SbCslH3* [28, 29, 38], were measured by quantitative PCR, Arun pith had higher transcript levels of *SbCslF6* and *SbCslH3* than did Rio (Fig 5).

**Fig 6:**
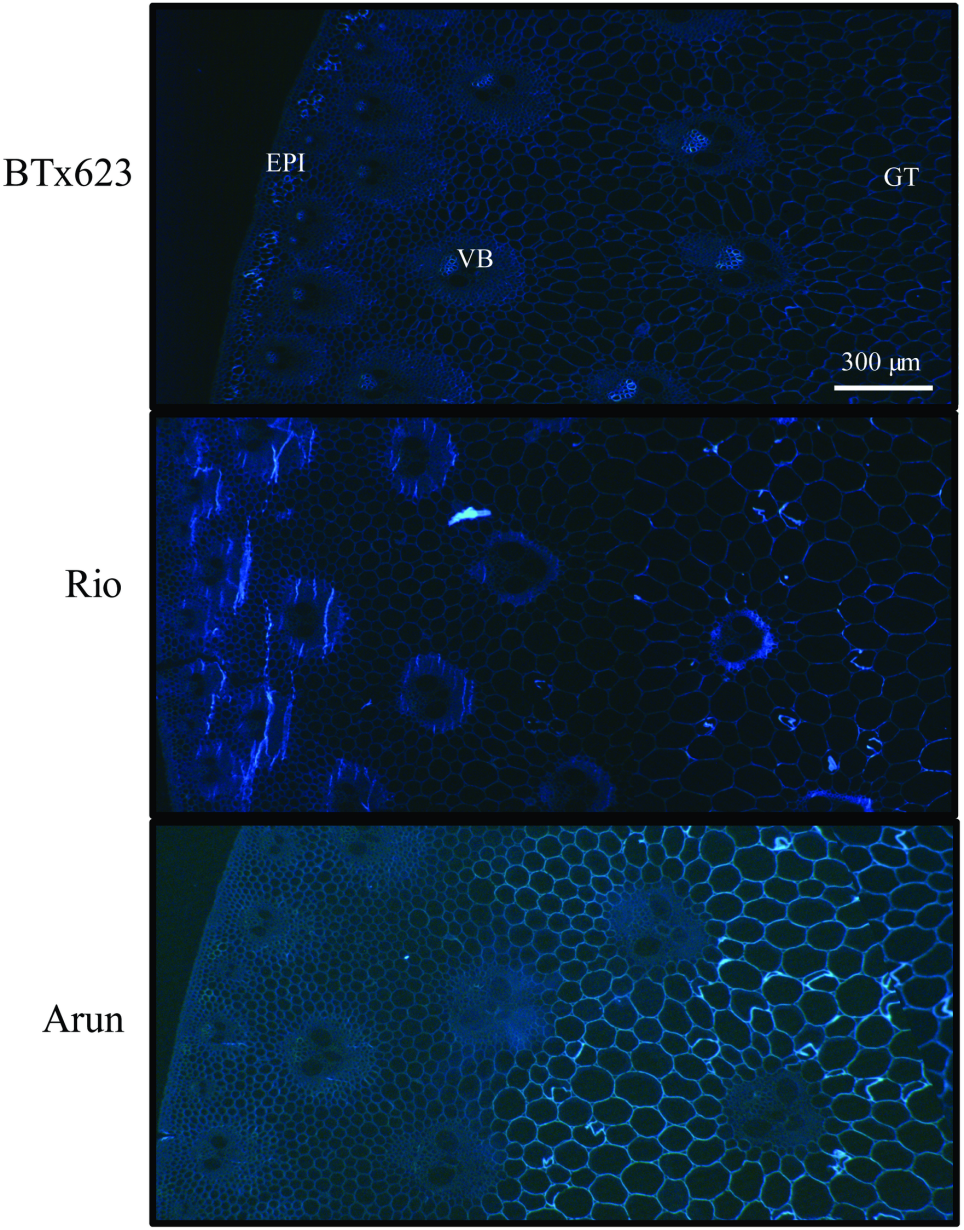
Distribution of glucan polymers in stems of grain, sweet and wild sorghum lines. Sections of mature sorghum stem were stained with Calcofluor-white and images captured with a dissection microscope and a fluorescence filter. All pictures are of the same scale, bar is 300 μm. Ground tissue (GT), vascular bundle (VB), epidermis (EPI).

The distribution of all β-(1,4)-linked glucans, such as cellulose and (1,3;1,4)-β-glucan, was observed by fluorescence of transverse tissue sections after staining with Calcofluor-white (Fig 6). BTx623 and Rio displayed a similar staining pattern, with fluorescence visible predominantly in tissues such as the phloem and in patches of parenchyma located in the rind. Arun however, showed bright fluorescence in cell walls throughout the pith and rind (Fig 6).

**Fig 7:**
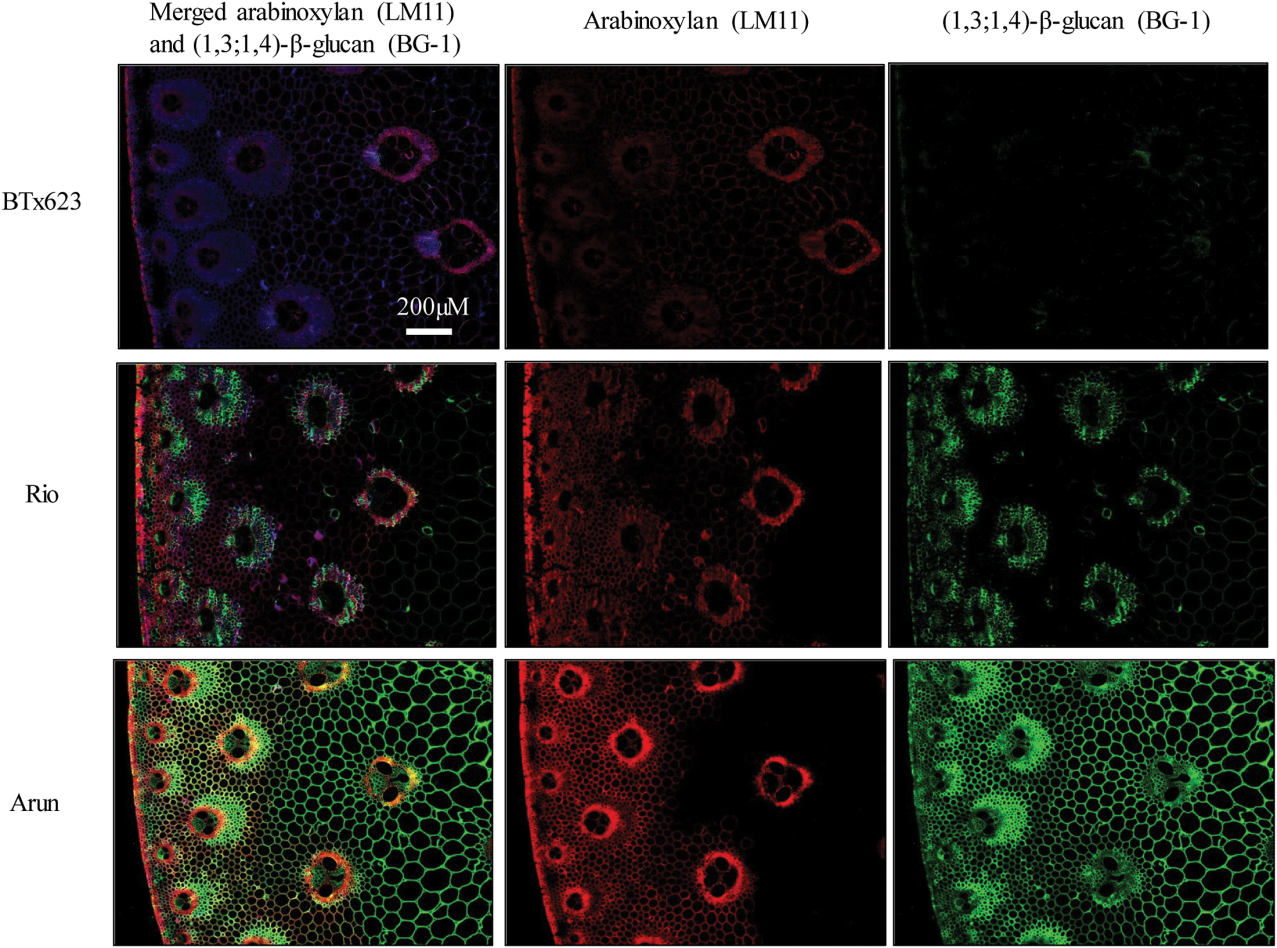
Distribution of (1,3;1,4)-β-glucan and arabinoxylan in sorghum stem sections. Immunohistological analysis of cell wall epitopes in BTx623, Rio and Arun stems. All panels show transverse sections through mature stems. In merged and single images the (1,4)-β-xylan/arabinoxylan labelled with an LM11 antibody is indicated by red fluorescence and the (1,3;1,4)-β-glucan, labelled with the BG-1 antibody is indicated by green fluorescence. All pictures are of the same scale, bar is 200 μm.

The specific distribution of arabinoxylan and (1,3;1,4)-β-glucan was investigated by labelling fixed stem tissues with fluorescent antibodies against these polysaccharides (Fig 7). The relative fluorescence associated with (1,3;1,4)-β-glucan labelling was consistent with the quantitative data (Fig 4d). The variation in fluorescence associated with LM11 between genotypes did not correlate well with the variation in the quantitative data for the sum of arabinose and xylose in each genotype (Fig 4c). However, sections labelled with the LM11 anti-arabinoxylan antibody had brighter fluorescence in rind tissue than pith tissue, and this was consistent with the quantitative data; and pith sections labelled with the BG-1 anti-(1,3;1,4)-β-glucan antibody had brighter fluorescence than rind tissue, consistent with the quantitative data (Fig 4).

### Starch and Glucose Yields from Sorghum Stems

**Fig 8:**
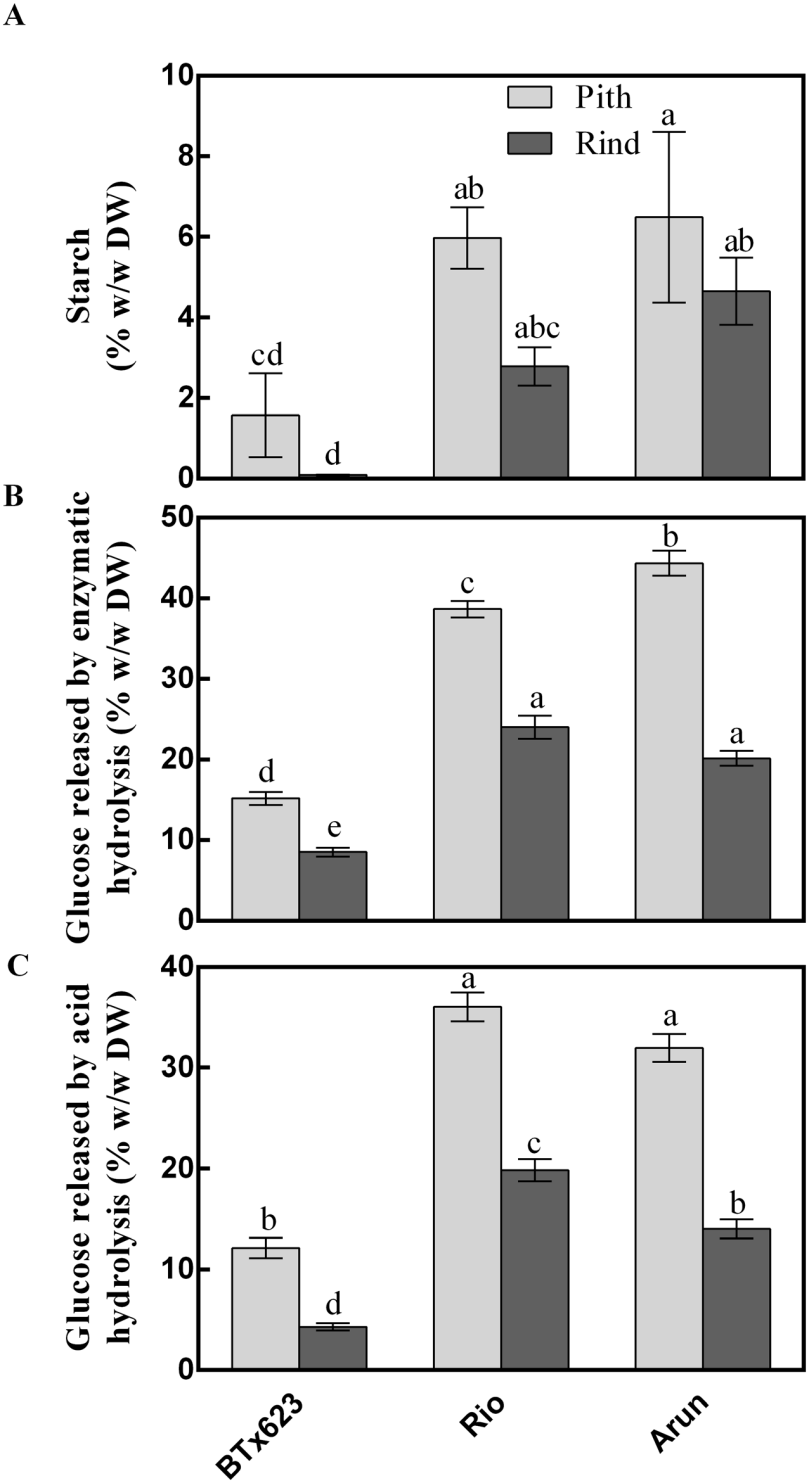
Sorghum stem starch amounts and relative digestibility. (**a**) Total starch in sorghum stem tissues; (**b**) glucose released after enzymatic digestion; and (**c**) glucose released after sulphuric acid digestion. Mean and standard error of three biological replicates; three technical replicates per assay. Different letters indicate statistical differences (ANOVA details in S3 Table).

Rio and Arun contained similar amounts of starch in their pith and rind, while BTx623 pith contained much less, with less even still in the rind (Fig 8a). The digestibility of stem samples was investigated by measuring the amount of glucose in the samples after either a commercial cellulase enzyme treatment or a sulfuric acid treatment. The enzyme treatment was more effective than the acid at liberating glucose for all tissues tested, especially the Arun samples (Fig 8b and 8c). With enzymatic digestion, BTx623 pith yielded 15% w/w glucose, while the rind yielded only 9% w/w. Rio biomass released 39% w/w glucose from the pith and 24% w/w from the rind. The highest glucose yield was from Arun pith, which yielded 44% w/w glucose after enzymatic pre-treatment, while rind glucose yields were much lower, at 20% w/w.

### Theoretical Ethanol Yields from Sorghum Stems

BTx623, Rio and Arun stem biomass and monosaccharide composition data (S1 and S2 Tables) were used to calculate the theoretical ethanol yields following the National Renewable Energy Laboratory Theoretical Ethanol Yield Calculator method (U.S. Department of Agriculture 2013; Table 1). The values represent estimates for stem tissue only; leaf and grain head biomass was not included. If the sugar in harvested stem tissue were efficiently used to produce ethanol, the estimates indicate that up to 10344 L/ha may be produced from Arun stem tissues.

**Table 1:** Theoretical ethanol yields from stem tissue of wild, sweet and grain sorghum.

	Theoretical ethanol yields from stem tissue		
Sorghum	L/Kg	L/plant stem	*L/ha
BTx623	0.20	0.004	728
Rio	0.28	0.033	5969
Arun	0.28	0.057	10344

Calculated using the National Renewable Energy Laboratory Theoretical Ethanol Yield Calculator (http://www1.eere.energy.gov/bioenergy/ethanol_yield_calculator.html) that assumes 1.111 Kg monomeric C6 sugar per 1 Kg polymeric C6 sugar polymer; 1.1363 Kg monomeric C5 sugar per 1 Kg polymeric C5 polymer; 0.51 Kg ethanol produced from 1 Kg sugar; 0.7892 g/L relative density of ethanol. Calculations are for dry weight and composition of mature stem tissue only and do not include ethanol that could be produce from leaf or grain tissues.

*Estimated based on a standard planting density of 180 000 plants/ha and stem dry weight distribution of 1:1 pith:rind. Values for sorghum stem monosaccharide composition used for the calculations are listed in S1 Table.

## Discussion

### (1,3;1,4)-β-Glucan in Cell Walls of Sorghum Stems

The stems of sorghum varieties investigated in this study varied in the amounts and composition of cell walls in pith and rind tissues (Figs 1 and 4) although no morphological differences could be observed (Fig 3b). The outer rind was generally comprised of higher amounts of cellulose and arabinoxylan compared with the inner pith, which contained higher amounts of (1,3;1,4)-β-glucan (Figs 1, 4, 6 and 7). These data are consistent with the roles played by these stem tissues. The rind has a more structural role, providing rigidity and support for the whole plant and housing the vascular bundles, so it is not surprising that cell walls here consist of molecules such as cellulose and arabinoxylan associated with greater recalcitrance [17–19]. In contrast, the pith has more of a storage role, accumulating excess carbohydrates that buffer source-sink interactions during growth in varying environmental conditions and contributing to plasticity in sink-source dynamics [39]. Storing a carbohydrate buffer in the stem, away from photosynthetic tissues, may also prevent feedback inhibition of photosynthesis, which has been observed when carbohydrates accumulate in the leaves [40, 41].

(1,3;1,4)-β-Glucan may accumulate in cell walls so that it can be readily degraded to release glucose for plant growth and reproduction [42, 43]. There is limited information about the importance of (1,3;1,4)-β-glucan as a storage polysaccharide in grasses and how the accumulation of (1,3;1,4)-β-glucan in vegetative tissues influences plant fitness and survival [44]. It is possible that the (1,3;1,4)-β-glucan in sorghum stem tissue is remobilized after the main tillers have reached maturity to supply glucose for the rapid growth of new tillers from the stalk base, the ratoon crop.

Sorghum varieties that are sensitive to photoperiod and do not flower under long day lengths are capable of achieving high levels of biomass production by continuing vegetative growth throughout the season [45]. These sorghum varieties tend to have high yields of lignocellulosic material and some lines produce 80 Mg ha^-1^ (65% moisture; [12]). Four photoperiod-sensitive lines grown as part of the current study had five times more (1,3;1,4)-β-glucan in the stem pith and rind relative to the photoperiod-insensitive lines that flowered (Fig 1a). The photoperiod-sensitive lines may have partitioned excess photoassimilate, not required for producing seed, into the production of (1,3;1,4)-β-glucan in the stem.

Sorghum lines that preferentially store stem glucose as (1,3;1,4)-β-glucan rather than as sucrose, as in sweet sorghum stems, may have improved post-harvest storage characteristics [46] and higher yields of fermentable carbohydrate [47]. Germplasm with high amounts of (1,3;1,4)-β-glucan in the stem, such as the ssp. *verticilliflorum* line identified here, could be incorporated into breeding programs to improve storage and conversion traits for biofuel production. Alternatively, it may be possible to overexpress genes involved in the synthesis of (1,3;1,4)-β-glucan in sorghum [48]. Previously, overexpression of a (1,3;1,4)-β-glucan synthesis gene (*CslF6*) in barley (*Hordeum vulgare*) resulted in sixfold higher amounts of (1,3;1,4)-β-glucan in the leaves but a significant reduction in plant fitness [49]. Recently, senescence-associated promoters were used to overexpress *CslF6* in *Arabidopsis*, resulting in a 42% increase in saccharification without affecting plant health [47]. Another strategy may be to identify sorghum lines with mutations in (1,3;1,4)-β-glucan degradation; for example, elevated levels of (1,3;1,4)-β-glucan were observed in a maize plant that had a defective lichenase [50].

Previous research in barley, wheat and rice has indicated that two types of *Csl* genes, *CslF* and *CslH*, are associated with the synthesis of the non-cellulosic polysaccharide (1,3;1,4)-β-glucan [28, 29, 47, 49, 51]. In sorghum, relatively high expression of *SbCslF6* has been observed in stem compared with other tissues, with an increase in *SbCslH3* expression as stem tissues mature [38]. The current data indicate that an increase in expression of *SbCslF6* and *SbCslH3* in mature stem tissues is associated with the accumulation of (1,3;1,4)-β-glucan (Figs 4d and 5b). Previous studies have suggested that glycan synthases may require the interaction of multiple proteins to translocate the polymer across the membrane [52], raising questions as to whether different CSL isoforms, such as CSLF and CSLH, interact with each other or with CESAs to form an integral catalytic complex. The identification of other factors that influence the regulation of (1,3;1,4)-β-glucan synthesis is also a key target for further research.

### Biofuel Production and Genetic Variation in Biomass Yield and Composition

High yields of bioethanol can be produced from sorghum [53]. For example the calculated ethanol yield from the late maturing sweet sorghum ‘Lvneng-3’, which had particularly high yields of cellulose and non-cellulosic polysaccharides, was up to 13 032 L ha^-1^ [54]. Yields are dependent not only on the amount of fermentable sugar in the plant, but also on the presence, amount and types of inhibitory molecules, such as lignin and ash, and the total amount of biomass produced per plant [55].

A detailed examination of three different types of sorghum varieties—a grain, sweet and wild line—revealed that the plants produce very different amounts of stem, leaf and grain tissues (Fig 2a and S2 Table). The grain line, BTx623, bred to have a shorter stature with high yields of grain, channelled 56% of biomass production to the grain head. Conversely, the sweet line, Rio, bred for long stems full of sucrose, partitioned 59% of its biomass into stem tissue. However, the uncultivated wild Arun line produced the most biomass (321g dry biomass per plant) with 65% biomass in the stem.

The starch content of Arun and Rio stem tissues did not differ, whereas there was more glucose released by enzymatic hydrolysis from Arun than Rio indicating that starch was not the likely source of the difference in glucose (Fig 8). Arun accumulated significantly higher levels of starch in the stem than BTx623, however on a % w/w basis the proportional difference in starch content would be insufficient to account for the proportional differences in glucose released (Fig 8). As starch is readily convertible to glucose during pre-treatment, engineering sorghum stems to increase starch accumulation may be a valid target for the improvement of sorghum as a biofuel crop or industrial raw material. Pre-treating stem tissues from these three varieties either with an enzyme cocktail or with sulfuric acid released up to 44% w/w glucose. The highest yield was from the Arun pith after enzymatic treatment (Fig 8b) with the lower yield from Arun rind (20% w/w) possibly due to the rind having the highest levels of lignin present in the three lines (Fig 4a). However, it is not clear whether lignin content is a factor that influences the release of glucose from (1,3;1,4)-β-glucan during enzymatic treatment.

Data for stem mass and composition (S1 and S2 Tables) were used to calculate theoretical ethanol yields from the stems of the three lines. Previous estimates from whole BTx623 and Rio plants ranged up to 5115 L ha^-1^ and the yield of hybrid plants was up to 8512 L ha^-1^ [15]. The values obtained in this study were very similar for Rio (5977 L ha^-1^), but much lower for BTx623 (721 L ha^-1^), presumably because we assessed stem tissue only, which comprises only 22% of the biomass of this grain variety.

The calculated values for the wild Arun stem, 10 344 L ha^-1^, were greater than the values for most high-yielding recombinant inbred lines from a BTx623 × Rio cross [15]. Recent research in rice indicates that yield from greenhouse-grown plants was a suitable predictor for yield in the field [56], so our results may translate to field-grown crops but future field trials to test this are needed. *S*. *bicolor* ssp. *verticilliflorum* is a weedy, wild relative of sorghum that grows on roadsides [9], so is an excellent candidate for successful cultivation in remote or marginal areas with little or no production inputs.

The cost of producing biofuel may become increasingly competitive with petroleum fuels if the current trajectory of genetic improvement in bioenergy sorghum continues [57] but it does depend on accurately identifying variation in morphological, structural and physiological traits [36]. Plant size is the main trait influencing bioenergy yield and more effort has so far been invested in increasing overall plant size than in improving the cell wall composition, which is more difficult to screen for in the field [36, 58]. However, the development of new high-throughput methods for screening biomass composition [32] combined with the emerging genomics resources available to the sorghum community [2, 5], make it increasingly feasible to select for genetic variation in structural and physiological traits that enhance biofuel production.

Exploring the diversity in sorghum biomass cell wall composition is important because natural genetic variation will provide the foundation for the improvement of sorghum vegetative tissues as an energy crop [59]. The biomass digestibility and carbohydrate yields from sorghum varieties are linked to the quality and quantity of carbohydrates, for example starch and (1,3;1,4)-β-glucan [45]. The literature currently lacks information about such variation in starch and (1,3;1,4)-β-glucan in diverse sorghum germplasm. Here, we have identified a wild, photoperiod-sensitive sorghum line that accumulates 6.5% starch and 5.5% w/w (1,3;1,4)-β-glucan in the stem pith, relative to the 1% w/w (1,3;1,4)-β-glucan observed in many cultivated varieties. This wild *S*. *bicolor* ssp. *verticilliflorum* had significantly higher stem digestibility and calculated ethanol yields relative to grain sorghum BTx623 despite having double the lignin content, at theoretical yields of 10344 L ha^-1^. This heterogeneity in glycan polymers in sorghum stems can be exploited to improve sorghum breeding for forage and biofuel industries.

## Materials and Methods

### Plant Materials

All 12 sorghum genotypes (listed in S1 Table) were grown in the Plant Accelerator (Australian Plant Phenomics Facility, Waite Campus, South Australia, Australia) in glass houses under a natural day length 30°C day temperature and 20°C night temperature in October 2011– February 2012 (day length 13:28 h to 12:53 h, respectively). Eight photoperiod-insensitive lines (Rio, Acme Broomcorn, LR2490-3, SC170-6-8, IS8525, BTx623, QL12 and M35-1) were harvested at maturity, 129 d after planting. Ssp. *verticilliflorum* (Arun) is partially photoperiod-sensitive, thus flowering was delayed and this line was harvested 159 d after planting. Three photoperiod-sensitive lines *S*. *drummondii*, Kinto Oule (PI525695) and PI559871 did not flower and these lines were harvested 248 d after planting. The third internode from the base of the plants was harvested, split manually into pith and rind sections and snap frozen in liquid nitrogen or placed into fixative for histological analysis. To determine plant height, each tiller was measured from the soil surface to the panicle tips. The mean stem diameter of each line was calculated by measuring the diameter at the first internode above the ground and averaging the tillers. For fresh biomass yield, the leaf, head and stem of each line were weighed immediately after harvest and dried at 60°C to constant moisture content. Samples were re-weighed to determine the dry biomass yield.

### Biomass Compositional Assays and Digestibility Assays

Tissue for all analyses was harvested, snap frozen in liquid nitrogen and freeze-dried (Labconco Freezone; Adelab Scientific, Adelaide, SA, Australia). Dried material was ground in a 25 mL stainless steel grinding jar with one 7 mm steel ball (Retsch mill MM400, Retsch GmbH; Haan, Germany).

#### Acid determination of cellulose

This method was adapted from [60] and [61] as follows; ground dried tissue (20 ± 1 mg) was treated with a mixture (1 mL) of acetic and nitric acid (8:1) in water at 110°C in heating blocks. Samples were washed once with water and twice with ethanol (80%), followed with an acetone (100%) wash before drying to a constant weight in a 45°C oven. The remaining material was recorded as acid-insoluble cellulose. Two technical replicates and three biological replicates were assayed per sample.

#### (1,3;1,4)-β-glucan analysis

This method and analysis followed [38]. Two technical replicates and three biological replicates were assayed per sample.

#### Starch assay

A small scale version of the Total Starch Assay procedure (AA/AMG method; Megazyme, Ireland) was used on 20 mg flour. The liberated glucose content was quantified using a spectrophotometer (Thermo Fischer, Waltham, MA, USA) at a wavelength of 510 nm. Samples were blanked with water and a maize starch standard (97%) was included in each batch. Two technical replicates and three biological replicates were assayed per sample.

#### Monosaccharide (mannose, ribose, rhamnose, glucuronic acid, galacturonic acid, glucose, galactose, xylose and arabinose) profiling of sorghum stems

Acid hydrolysis and reversed-phase high-performance liquid chromatography (RP-HPLC) methodology followed [62, 63]. A 20 mg amount of freeze dried tissue was washed four times with 70% ethanol to remove soluble sugars, acid hydrolysed in 1 M sulfuric acid at 100°C for 3 h and analysed by RP-HPLC as described by [64]. Three biological replicates and three technical replicates were used to assess the variation in monosaccharide content.

#### Klason lignin

The method for assaying lignin followed [65] with the following modifications; each 20 mg tissue sample were washed twice with ethanol (80%) and placed at 45°C overnight prior to 12 M sulfuric acid treatment at 35°C in 10 mL glass tubes. Acid-soluble contents were removed from the tissues by washing three times with 10 mL of water. The pellets were diluted in water, filtered through Whatman GF/C55mm glass microfiber filters (Sigma-Aldrich, St Louis, USA) and dried to a constant weight in a 45°C oven. The remaining material was recorded as acid-insoluble lignin. Two technical replicates and three biological replicates were assayed per sample.

#### Stem digestibility

The method for assaying digestibility followed [66] with the following modifications; a 25 mg aliquot of ground dried tissue was weighed into 10 mL glass tube and sterilized at 121°C for 20 min with water to prevent bacterial contamination and disrupt cell wall structure. The solution was treated with an enzyme mixture (C2730; Sigma-Aldrich) containing cellulase from *Trichoderma reesei* (≥ 700 units/g) and cellobiase from *Aspergillus niger* (≥ 250 units/g), at 50°C for 72 h to hydrolyze glucans into sugars. The enzyme mix also included a (1,3;1,4)-β-glucanase. The reaction was diluted and incubated with glucose oxidase/peroxidise reagent (GOPOD; Megazyme International, Ireland) at 50°C for 20 min. The absorbance was read at 510 nm using a spectrophotometer (Thermo Fischer) along with a glucose standard and a blank of GOPOD and enzyme mixture. Glucose released from stem tissues by 1 M sulfuric acid was quantified following [53]. Two technical replicates and three biological replicates were assayed per sample.

#### Theoretical ethanol yields

These were calculated following [67] using the following standard conversion assumptions; 1.111 kg monomeric C6 sugar per 1 kg polymeric C6 polymer (glucan, fructan); 1.1363 kg monomeric C5 sugar per 1 kg polymeric C5 polymer (xylan, arabinan); 0.51 kg of ethanol produced from 1 kg of sugar; 0.7892 g/L relative density of ethanol.

### Histological Analysis and Immunolabelling

Histological analysis followed [62]: Replicate stem segments were harvested at maturity and fixed overnight in formalin–acetic acid–alcohol (FAA) solution containing 50% ethanol, 5% acetic acid and 4% formaldehyde. Samples were serially dehydrated and embedded in LR White resin (London Resin Co., UK) as described by [49]. Sections, 500 nm thick, were cut using a Reichert Ultracut ultramicrotome (Leica, Germany). For immunolabelling, sections were labelled with the (1,4)-β-xylan/arabinoxylan specific rat monoclonal antibody LM11 (McCartney, Marcus & Knox 2005; PlantProbes, Leeds, UK) and the (1,3;1,4)-β-glucan specific mouse primary antibody BG-1 ([68]; Biosupplies, Australia). Secondary antibodies were conjugated to either Alexafluor 488 or 555 (Life Technologies, USA) and samples were examined using a Zeiss M1 AxioImager. Maximum fluorescence intensity measurements were generated using ZEN 2012 software, as described by [69]. When sorghum sections were treated with a lichenase enzyme (Megazyme), (1,3;1,4)-β-glucanase associated fluorescence disappears confirming specificity of (1,3;1,4)-β-glucan labelling [28]. Calcofluor White (Sigma-Aldrich) was used as a general counter-stain to detect β-linked glucose polymers where sections were incubated with 0.1% Calcofluor White and images of labeled sections were taken under fluorescence filter I3 (450–490 nm BP, barrier 515 nm LP) and filter D (335–425 nm BP, barrier 470 nm LP) using a Leica AS LMD Laser Dissection Microscope with a DFC 480 camera.

### Quantitative Polymerase Chain Reaction (Q-PCR)

Total RNA was extracted from mature stems using the Direct-zol RNA MiniPrep (Zymo Research, USA). cDNA was synthesized according to [70]. Data is from three biological replicates. Q-PCR analysis and data normalisation using multiple control genes (glyceraldehyde-3-phosphate dehydrogenase (GAPDH), α-tubulin, cyclophilin) followed [38]. Primer sequences are provided in S4 Table.

### Statistical Analysis

Analysis of variance (ANOVA) and Tukey’s multiple comparison tests were undertaken using GENSTAT (VSN International) or GraphPad Prism (GraphPad Software Inc., USA). Statistical information is included in S3 Table.

## Supporting Information

S1 Table Genotype, photoperiod sensitivity and cell wall composition data for twelve diverse sorghum lines. (DOC)

S2 Table Morphological and biomass traits for representative grain, sweet and wild sorghum lines. (DOC)

S3 Table Summary of statistics. (DOC)

S4 Table Quantitative PCR primers and product sizes. (DOC)
