# Supplementary material for: Prospecting for Energy-Rich Renewable Raw Materials: Sorghum Stem Case Study
Source: PLoS One. 2016 May 27;11(5):e0156638. doi: 10.1371/journal.pone.0156638 (PMC4883800; doi:10.1371/journal.pone.0156638)
Supplement: S1 Table — (DOC) [file pone.0156638.s001.doc]

S1 Table. Genotype, photoperiod sensitivity and cell wall composition data for twelve diverse sorghum lines. Mean and standard error (SE) for three biological replicates with two technical replicates per assay. ^*^ *S. bicolor* subsp. *verticilliflorum* (Steud., De Wet ex Wiersema & J. Dahlb.; previously classified as *S. arundinaceum*; Arun).Values given in % w/w of dry weight.

| **Sorghum genotype** | | | **Cellulose** | | | | **(1,3;1,4)-β-glucan** | | | | **Arabinose and xylose** | | | |
| --- | --- | --- | --- | --- | --- | --- | --- | --- | --- | --- | --- | --- | --- | --- |
| **Genotype** | **AusTRCF number** | **Photoperiod** | **pith** | **SE** | **rind** | **SE** | **pith** | **SE** | **rind** | **SE** | **pith** | **SE** | **rind** | **SE** |
| PI559871 | AusTRCF303220 | Sensitive | 9.5 | 0.4 | 24.9 | 4.9 | 4.6 | 0.4 | 1.2 | 0.1 | 6.0 | 0.6 | 15.7 | 0.5 |
| KintoOule (PI525695) | AusTRCF322646 | Sensitive | 16.9 | 2.9 | 25.2 | 7.5 | 4.9 | 0.5 | 1.7 | 0.2 | 11.7 | 0.5 | 15.4 | 0.3 |
| ssp. *drummondii* | AusTRCF300236 | Sensitive | 18.6 | 1.6 | 28.4 | 14.7 | 5.6 | 0.2 | 2.8 | 0.1 | 9.0 | 0.2 | 17.1 | 0.3 |
| ssp. *verticilliflorum** | AusTRCF317961 | Partially sensitive | 20.9 | 3.4 | 31.6 | 10.4 | 5.5 | 0.1 | 2.0 | 0.5 | 9.8 | 0.3 | 15.3 | 0.3 |
| Rio | AusTRCF95697 | Insensitive | 7.8 | 0.6 | 23.4 | 4.9 | 0.9 | 0.4 | 0.5 | 0.1 | 4.6 | 0.7 | 13.7 | 0.4 |
| Acme Broomcorn | AusTRCF313580 | Insensitive | 18.3 | 2.6 | 34.0 | 4.8 | 2.1 | 0.3 | 0.4 | 0.1 | 16.3 | 0.3 | 15.4 | 0.3 |
| LR2490-3 | AusTRCF304132 | Insensitive | 20.2 | 1.9 | 32.3 | 8.2 | 0.8 | 0.8 | 0.3 | 0.3 | 14.3 | 0.4 | 16.4 | 2.1 |
| SC170-6-8 | AusTRCF314392 | Insensitive | 18.8 | 2.0 | 29.1 | 6.4 | 1.0 | 0.4 | 0.5 | 0.1 | 14.5 | 0.2 | 20.2 | 1.4 |
| IS8525 | AusTRCF101487 | Insensitive | 19.4 | 3.0 | 31.4 | 12.2 | 1.7 | 0.5 | 0.3 | 0.1 | 9.7 | 0.3 | 16.4 | 0.3 |
| BTx623 | AusTRCF312557 | Insensitive | 17.1 | 1.1 | 35.3 | 5.6 | 0.2 | 0.1 | 0.1 | 0.1 | 12.3 | 0.2 | 20.5 | 0.1 |
| QL12 | AusTRCF313833 | Insensitive | 12.7 | 1.3 | 24.4 | 8.9 | 1.5 | 0.7 | 0.5 | 0.2 | 8.7 | 0.1 | 17.9 | 0.3 |
| M35-1 | AusTRCF316433 | Insensitive | 18.2 | 1.2 | 32.1 | 8.1 | 0.5 | 0.3 | 0.1 | 0.0 | 12.1 | 0.3 | 19.3 | 1.1 |
