# Supplementary material for: Prospecting for Energy-Rich Renewable Raw Materials: Sorghum Stem Case Study
Source: PLoS One. 2016 May 27;11(5):e0156638. doi: 10.1371/journal.pone.0156638 (PMC4883800; doi:10.1371/journal.pone.0156638)
Supplement: S2 Table — (DOC) [file pone.0156638.s002.doc]

S2 Table. Morphological and biomass traits for the selected grain, sweet and wild sorghum lines.

| **Trait** | **BTx623** | **Rio** | **Arun** |
| --- | --- | --- | --- |
| Height (max) | 124 cm | 239 cm | 237 cm |
| Tiller diameter (max) | 17 mm | 14 mm | 8 mm |
| Tiller number (max) | 1 | 2 | 13 |
| Plant fresh weight (mean and SEM) | 270 ± 12 g | 621 ± 14 g | 839 ± 91 g |
| Plant dry weight (mean and SEM) | 92 ± 5 g | 201 ± 14 g | 321 ± 21 g |
| Water content | 66% | 68% | 63% |
