# Supplementary material for: Prospecting for Energy-Rich Renewable Raw Materials: Sorghum Stem Case Study
Source: PLoS One. 2016 May 27;11(5):e0156638. doi: 10.1371/journal.pone.0156638 (PMC4883800; doi:10.1371/journal.pone.0156638)
Supplement: S3 Table — (DOC) [file pone.0156638.s003.doc]

S3 Table. Summary of statistics

| **Figure details:** | **Figs. 4B, C, D** | | | **Figs. 8A, 8B, 8C, 8D** | | | **Figs. 3A, 4A** | | |
| --- | --- | --- | --- | --- | --- | --- | --- | --- | --- |
| **Sorghum line and tissue sample** | **Cellulose** | **BG** | **AX** | **Starch** | **Enzymatic hydrolysis** | **Acid hydrolysis** | **Sample**  **(whole stem)** | **Klason lignin** | **AIR** |
| Arun pith vs. Arun rind | ns | **** | **** | ns | **** | **** | Arun vs. Rio | **** | **** |
| Arun pith vs. Rio pith | * | **** | **** | ns | * | ns | Arun vs. BTx623 | ** | ** |
| Arun pith vs. Rio rind | ns | **** | ** | ns | **** | **** | Rio vs. BTx623 | ns | * |
| Arun pith vs. BTx623 pith | ns | **** | ns | * | **** | **** |  |  |  |
| Arun pith vs. BTx623 rind | * | **** | **** | ** | **** | **** | **KEY** | | |
| Arun rind vs. Rio pith | *** | ** | **** | ns | **** | **** | **P Value summary** | | |
| Arun rind vs. Rio rind | ns | *** | ns | ns | ns | ** | **< 0.0001** | ******** | |
| Arun rind vs. BTx623 pith | * | **** | * | ns | * | ns | **< 0.001** | ******* | |
| Arun rind vs. BTx623 rind | ns | **** | **** | * | **** | **** | **< 0.01** | ****** | |
| Rio pith vs. Rio rind | ** | ns | **** | ns | **** | **** | **< 0.05** | ***** | |
| Rio pith vs. BTx623 pith | ns | ns | **** | * | **** | **** | Not significant | ns | |
| Rio pith vs. BTx623 rind | **** | ns | **** | *** | **** | **** |  |  | |
| Rio rind vs. BTx623 pith | ns | ns | ns | ns | **** | *** |  |  |  |
| Rio rind vs. BTx623 rind | ns | ns | **** | ns | **** | **** |  |  |  |
| BTx623 pith vs. BTx623 rind | ** | ns | **** | ns | ** | *** |  |  |  |
