# Supplementary material for: Prospecting for Energy-Rich Renewable Raw Materials: Sorghum Stem Case Study
Source: PLoS One. 2016 May 27;11(5):e0156638. doi: 10.1371/journal.pone.0156638 (PMC4883800; doi:10.1371/journal.pone.0156638)
Supplement: S4 Table — (DOC) [file pone.0156638.s004.doc]

S4 Table. Q-PCR primers and PCR product sizes. *Control genes (Ermawar et al. 2015)

| **Gene** | **Forward primer** | **Reverse primer** | **PCR product (bp)** | **Acquisition temperature (^o^C)** |
| --- | --- | --- | --- | --- |
| GAPSb^*^ | GATCAGTGACGCAATGGAGA | ATCTCAGCCCATGGTGTAGG | 196 | 83 |
| TubulinSb^*^ | GCGTGTGGTGGTGATGTTAG | TGCATAAGGCATAGCAACAA | 162 | 78 |
| CyclophilinSb^*^ | TTGCTAGTGGTCGTTTGTCG | GATGATGATGCGTGAGACCA | 151 | 78 |
| *SbCslF6* | CCAGCATCTATCGTCAAGCATGC | CTGTGTGGGGTTGCTGTGTAGTC | 159 | 81 |
| *SbCslH3* | CTTTCGCTGGTGTCAGAATATGC | CCAGCCAGATGCTAGTCGTCTTG | 174 | 78 |
